# Seroepidemiology of Coxsackievirus A6, Coxsackievirus A16, and Enterovirus 71 Infections among Children and Adolescents in Singapore, 2008-2010

**DOI:** 10.1371/journal.pone.0127999

**Published:** 2015-05-26

**Authors:** Li Wei Ang, Joanne Tay, Meng Chee Phoon, Jung Pu Hsu, Jeffery Cutter, Lyn James, Kee Tai Goh, Vincent Tak-Kwong Chow

**Affiliations:** 1 Epidemiology and Disease Control Division, Ministry of Health, Singapore, College of Medicine Building, 16 College Road, Singapore 169854, Singapore; 2 Communicable Diseases Division, Ministry of Health, Singapore, College of Medicine Building, 16 College Road, Singapore 169854, Singapore; 3 Department of Microbiology, Yong Loo Lin School of Medicine, National University Health System, National University of Singapore, MD4, 5 Science Drive 2, Singapore 117545, Singapore; 4 Saw Swee Hock School of Public Health, National University of Singapore, MD3, 16 Medical Drive, Singapore 117597, Singapore; University of Massachusetts Medical Center, UNITED STATES

## Abstract

Coxsackieviruses A6 (CV-A6) and A16 (CV-A16) and Enterovirus 71 (EV-A71) have caused periodic epidemics of hand, foot and mouth disease (HFMD) among children in Singapore. We conducted a cross-sectional study to estimate the seroprevalence of these enteroviruses among Singapore children and adolescents. The study was conducted between August 2008 and July 2010. It involved 700 Singapore residents aged 1–17 years whose residual sera were obtained following the completion of routine biochemical investigations in two public acute-care hospitals. The levels of neutralizing antibodies (NtAb) against CV-A6, CV-A16 and EV-A71 were analyzed by the microneutralization test. The age-specific geometric mean titer (GMT) of antibodies against each of the three enteroviruses and the 95% confidence intervals (CI) were calculated. The seroprevalence of CV-A6 and CV-A16 was high at 62.7% (95% CI: 59.1–66.2%) and 60.6% (95% CI: 56.9–64.1%), respectively. However, the seroprevalence of EV-A71 was significantly lower at 29.3% (95% CI: 26.0–32.8%). About 89.7% of the children and adolescents had been infected by at least one of the three enteroviruses by 13–17 years of age. About half (52.3%) were seropositive for two or all three enteroviruses, while only 16.1% had no NtAb against any of the three enteroviruses. High NtAb levels were observed in the younger age groups. CV-A6 and CV-A16 infections are very common among Singapore children and adolescents, while EV-A71 infections are less common. Infection is continually acquired from early childhood to adolescent age.

## Introduction

Coxsackieviruses A6 (CV-A6) and A16 (CV-A16) and Enterovirus 71 (EV-A71) are members of the *Enterovirus* genus of the family *Picornaviridae*. CV-A16 and EV-A71 are the most common etiologic agents of hand, foot and mouth disease (HFMD). HFMD is a common childhood viral infection characterized by a brief prodromal fever, followed by pharyngitis, rash with vesicles on the mouth, hands, feet and buttocks. It is typically mild and self-limiting.

Serious complications and fatalities related to HFMD tend to be associated more with EV-A71 than other enteroviruses such as CV-A16 [1]. This has triggered public health concerns about EV-A71 infection, especially in the Southeast and East Asian regions [2,3]. Since 1997, countries of the Asia Pacific Rim have been particularly affected by EV-A71-associated HFMD epidemics, such as Malaysia in 1997 [4], 2000 [5,6], 2003 [7], 2005 [8], and 2010 [9]; Brunei in 2006 [10]; Taiwan in 1998 [11] and 2000 [12]; Western Australia in 1999 [13], Japan in 1997 [14], 2000 and 2003 [15]; China in 2008 [16–18] and 2010 [19], Cambodia in 2012 [20], South Korea in 2000 [21] and 2008–2009 [22,23], and Vietnam in 2005 [24] and 2011–2012 [25].

HFMD is endemic in Singapore, with more than 50% of cases occurring in children below 5 years of age. It was made a notifiable disease under the Infectious Diseases Act on 1 October 2000 after a large EV-A71-associated HFMD outbreak which resulted in four pediatric deaths [26–28]. Nationwide HFMD epidemics were subsequently reported in 2002 (388.6 cases per 100,000 population) and annually since 2005 (ranging from 346.4 cases per 100,000 population in 2009 to 698.8 cases per 100,000 population in 2012). Excluding the epidemic years, the incidence per 100,000 population ranged from 125.4 in 2001 to 153.9 in 2004 [29]. Routine virologic surveillance of HFMD is carried out at two public acute-care hospitals with pediatric facilities (KK Women’s and Children’s Hospital [KKH] and National University Hospital [NUH]) and at a number of sentinel private general practitioner clinics. Such surveillance has shown that there are periodic changes in the predominant circulating enteroviruses causing HFMD. Since late 2009, the main circulating enterovirus associated with HFMD epidemics in Singapore has been CV-A6. Prior to that, the main enteroviruses causing nationwide HFMD epidemics were EV-A71 and CV-A16 [30,31].

**Fig 1:**
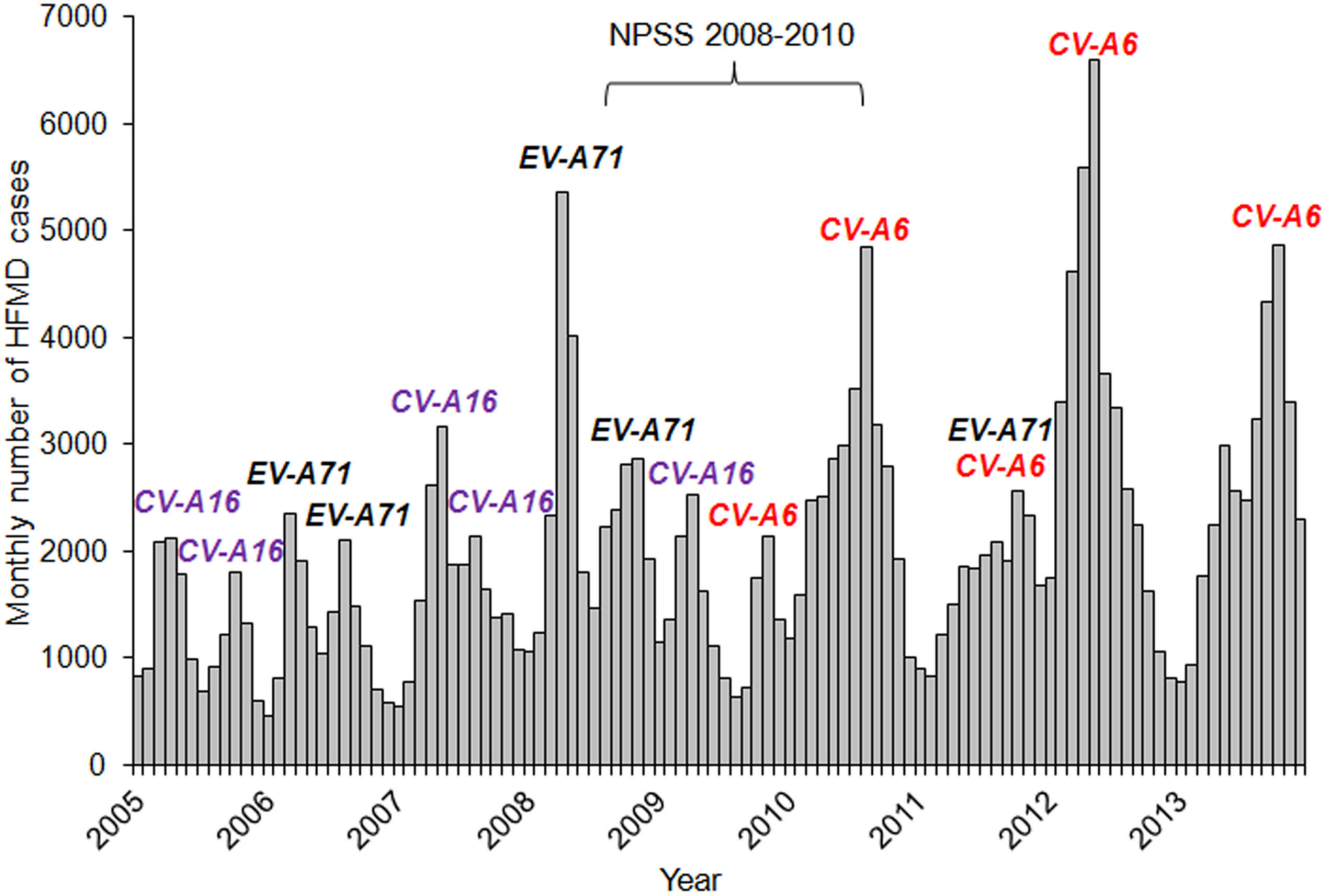
Monthly HFMD incidence and predominant circulating enteroviruses associated with peaks in HFMD cases, Singapore, 2005–2013.

We conducted a seroprevalence study among children and adolescents to estimate the prevalence of neutralizing antibodies (NtAb) against CV-A16, CV-A6 and EV-A71. Sera were collected between August 2008 and July 2010 for the National Paediatric Seroprevalence Survey (NPSS), when there were peaks in HFMD cases associated with these three enteroviruses (Fig 1).

## Materials and Methods

### Study design

The seroprevalence study on EV-A71 was incorporated in the NPSS on vaccine-preventable diseases conducted by the Ministry of Health (MOH) over a 24-month period between 2008 and 2010. The NPSS involved prospective collection of residual sera from children and adolescents following the completion of routine biochemical investigations by diagnostic laboratories in KKH and NUH [32]. About 79% of all hospitalized patients aged 1–17 years during the study period were admitted into these two hospitals.

The survey was carried out in accordance with Section 7 of the Infectious Diseases Act which provides for the use of residual blood samples for the purpose of public health surveillance. The residual sera for the survey were “leftover samples” collected for diagnostic purposes in KKH and NUH during hospitalization of the subjects, and no fresh blood samples were obtained for this study. Hence, the study did not pose any risk to human subjects as only their residual sera were used.

Residual sera of Singapore citizens and permanent residents who were ethnic Chinese, Malay and Indian aged 1–17 years attending inpatient services or day surgery were collected. Patients were excluded if they were known to be immunocompromised, on immunosupressive therapy, or had been diagnosed with HFMD, measles, mumps, rubella, chickenpox, diphtheria, pertussis, poliomyelitis, hepatitis B, or dengue. Residual sera from a total of 1,200 patients aged 1–17 years were prospectively collected for the NPSS until the target sample size of 400 in each of the three age groups, 1–6 years (pre-school), 7–12 years (primary school) and 13–17 years (secondary school), was attained. The age-ethnic distribution of the subjects by gender was comparable to that of the Singapore resident population aged 1–17 years in 2009 [33]. The personal identifiers were permanently deleted from the consolidated database containing their demographics.

For this study, residual sera from the NPSS were subsequently tested for CV-A6 and CV-A16 to assess the level of immunity of the pediatric population prior to the largest HFMD epidemic in 2012 when CV-A6 was the predominant enterovirus isolated. On the premise of an anticipated overall CV-A16 seroprevalence of 60% in children and adolescents aged 1–17 years, the minimum sample size required was 577, with a confidence level of 95% and an absolute precision of 4%. For logistic convenience, a total of 700 residual blood samples with sufficient amount of residual sera leftover from the NPSS 2008–2010 were used for the neutralization tests against CV-A16 and CV-A6. As this study was carried out under the Infectious Diseases Act for the purpose of public health surveillance, informed consent was not obtained from the parents or guardians on behalf of the children whose residual blood samples were included in the study. The proposed research including its protocol for the study was approved by the Institutional Review Board of the National University of Singapore (approval number: NUS 1832).

In this study comprising 700 subjects, there were almost three times as many subjects in each of the older age groups of 7–12 years and 13–17 years, compared to the youngest age group of 1–6 years (Table 1). The overall male-to-female ratio was 1:1.3. The ethnic distribution was similar to that of the NPSS 2008–2010.

**Table 1:** Demographic characteristics of 700 subjects aged 1–17 years, Singapore, 2008–2010.

Demographics		Number	Percent
Age group			
	1–6	104	14.9
	7–12	294	42.0
	13–17	302	43.1
Gender			
	Male	301	43.0
	Female	399	57.0
Ethnic group			
	Chinese	461	65.8
	Malay	165	23.6
	Indian	74	10.6

### Neutralizing antibody assays

The serum samples were stored at -80°C, membrane-filtered, and inactivated at 56°C for 30 minutes before use. A modified microneutralization test [34] was used for detecting neutralizing antibody against the three enteroviruses. EV-A71/5865/Sin/000009, a previously characterized EV-A71 subgenotype B4 strain isolated from a fatal case of encephalitis during the local HFMD epidemic in 2000 [35], CV-A6/NUH0026/SIN/08 obtained from a HFMD patient in Singapore during the local HFMD epidemic in 2008 [31], and CV-A16/G-10 prototype strain [36] were used to quantify NtAb levels against EV-A71, CV-A6 and CV-A16, respectively. Serum sample dilutions of 1:8 to 1:1,024 were assayed, and each dilution was tested in duplicate. 25 μl of 100 tissue culture infective dose (TCID_50_) of virus was mixed with 25 μl of the appropriate serum dilution, and incubated at 37°C for two hours in the presence of CO_2_, followed by the addition of 100 μl of RD rhabdomyosarcoma cell suspension (at 2 x 10^4^ cells per 0.1 ml). Infected cells and controls were incubated at 37°C in 5% CO_2_ and observed with an inverted microscope daily for four days. The highest dilution that prevented the development of cytopathic effect in 50% of the wells was considered the antibody titer of the sample. NtAb against CV-A6, CV-A16 and EV-A71 were considered positive if the dilution was ≥1:8. A positive serum sample of known antibody titer that was relatively reproducible, an antibody-negative serum sample, and uninfected cells were included as controls. A virus back titration was also performed to determine the virus titer, as well as to serve as “no serum” control.

### Statistical analyses

We calculated the 95% confidence intervals (CI) for binomial proportions using Wilson’s method [37]. The Chi-square test and Fisher’s exact test, where appropriate, were used to test the difference in seroprevalence between groups. The Chi-square test for trend was used to detect the presence of a linear trend across age groups.

The geometric mean titer (GMT) of positive sera and corresponding 95% CI were calculated by first taking the logarithmic transformation (base 2) of the titer readings, followed by antilog transformation of the mean and its 95% lower and upper confidence limits. For the computation of GMT, antibody titer with dilution >1:1,024 was assigned the value of 1,024. Comparison of GMT by age group was performed by first computing the mean and 95% CI of the difference in logarithm-transformed antibody titer. If there was no difference, the ratio of 1 would be within the confidence limits which had been antilog-transformed [38]. All *P* values reported were two-sided and statistical significance was taken at *P* < 0.05. Statistical analysis was performed using the statistical software package, SPSS Statistics version 19.0 (IBM, USA).

## Results

### Seroprevalence of enteroviral infections

In the 700 subjects aged 1–17 years, the seroprevalence of CV-A6 was the highest at 62.7% (95% CI: 59.1–66.2%), followed by CV-A16 at 60.6% (95% CI: 56.9–64.1%) (Table 2). The seroprevalence of EV-A71 was the lowest at 29.3% (95% CI: 26.0–32.8%). The EV-A71 seroprevalence based on a subset of 700 samples was similar to that based on 1,200 samples [26.9% (95% CI: 24.5–29.5%)] [32].

**Table 2:** Seroprevalence of CV-A6, CV-A16 and EV-A71 (with 95% confidence interval) by age group, gender and ethnic group among 700 subjects aged 1–17 years, Singapore, 2008–2010.

Demographics		CV-A6	CV-A16	EV-A71
All		62.7	60.6	29.3
		(59.1–66.2)	(56.9–64.1)	(26.0–32.8)
Age group (years)				
	1–6	51.9	50.0	15.4
		(42.4–61.3)	(40.6–59.4)	(9.7–23.5)
	7–12	63.6	52.0	26.2
		(58.0–68.9)	(46.3–57.7)	(21.5–31.5)
	13–17	65.6	72.5	37.1
		(60.0–70.7)	(67.2–77.2)	(31.8–42.7)
Gender				
	Male	66.1	58.8	27.6
		(60.6–71.2)	(53.2–64.2)	(22.8–32.9)
	Female	60.2	61.9	30.6
		(55.3–64.8)	(57.0–66.5)	(26.3–35.3)
Ethnic group				
	Chinese	59.7	57.0	26.2
		(55.1–64.0)	(52.5–61.5)	(22.4–30.4)
	Malay	67.3	70.3	38.2
		(59.8–74.0)	(62.9–76.7)	(31.1–45.8)
	Indian	71.6	60.8	28.4
		(60.5–80.6)	(49.4–71.1)	(19.4–39.5)

The CV-A6 seroprevalence increased significantly from 51.9% (95% CI: 42.4–61.3%) in the 1–6 year olds to 63.6% (95% CI: 58.0–68.9%) in the 7–12 year olds (*P* < 0.05), and remained stable at 65.6% (95% CI: 60.0–70.7%) in the 13–17 year olds. The CV-A16 seroprevalence in the two younger age groups was similar at 50.0% to 52.0%, and increased significantly to 72.5% (95% CI: 67.2–77.2%) in adolescents aged 13–17 years (*P* < 0.0005). The EV-A71 seroprevalence increased significantly with age (*P* < 0.0005); it rose from 15.4% (95% CI: 9.7–23.5%) in children aged 1–6 years to 26.2% (95% CI: 21.5–31.5%) in the 7–12 year olds and 37.1% (95% CI: 31.8–42.7%) in adolescents aged 13–17 years. This indicated that the EV-A71 seroconversion rate increased by about 11% between consecutive age groups.

The seroprevalence of EV-A71 was consistently the lowest in the three age groups compared to CV-A6 and CV-A16. In the age group of 7–12 years, the seroprevalence of CV-A6 was significantly higher than that of CV-A16 (*P* < 0.001).

There was no significant gender-specific difference for each of the three enteroviruses. There were no ethnic-specific differences in the CV-A6 seroprevalence among Chinese (59.7%), Malays (67.3%) and Indians (71.6%). Malays had significantly higher CV-A16 seroprevalence (70.3%) compared to Chinese (57.0%) (*P* < 0.005), while there was no significant difference compared to Indians (60.8%) (*P* = 0.18). There was also no significant difference in the CV-A16 seroprevalence between Chinese and Indians (*P* = 0.61). Similar to CV-A16, the EV-A71 seroprevalence in Malays (38.2%) was significantly higher than in Chinese (26.2%) (*P* < 0.005), while it was not significantly different from that of Indians (28.4%) (*P* = 0.15). There was no significant difference in the EV-A71 seroprevalence between Chinese and Indians (*P* = 0.78).

### Proportion of seropositivity by enterovirus type

**Fig 2:**
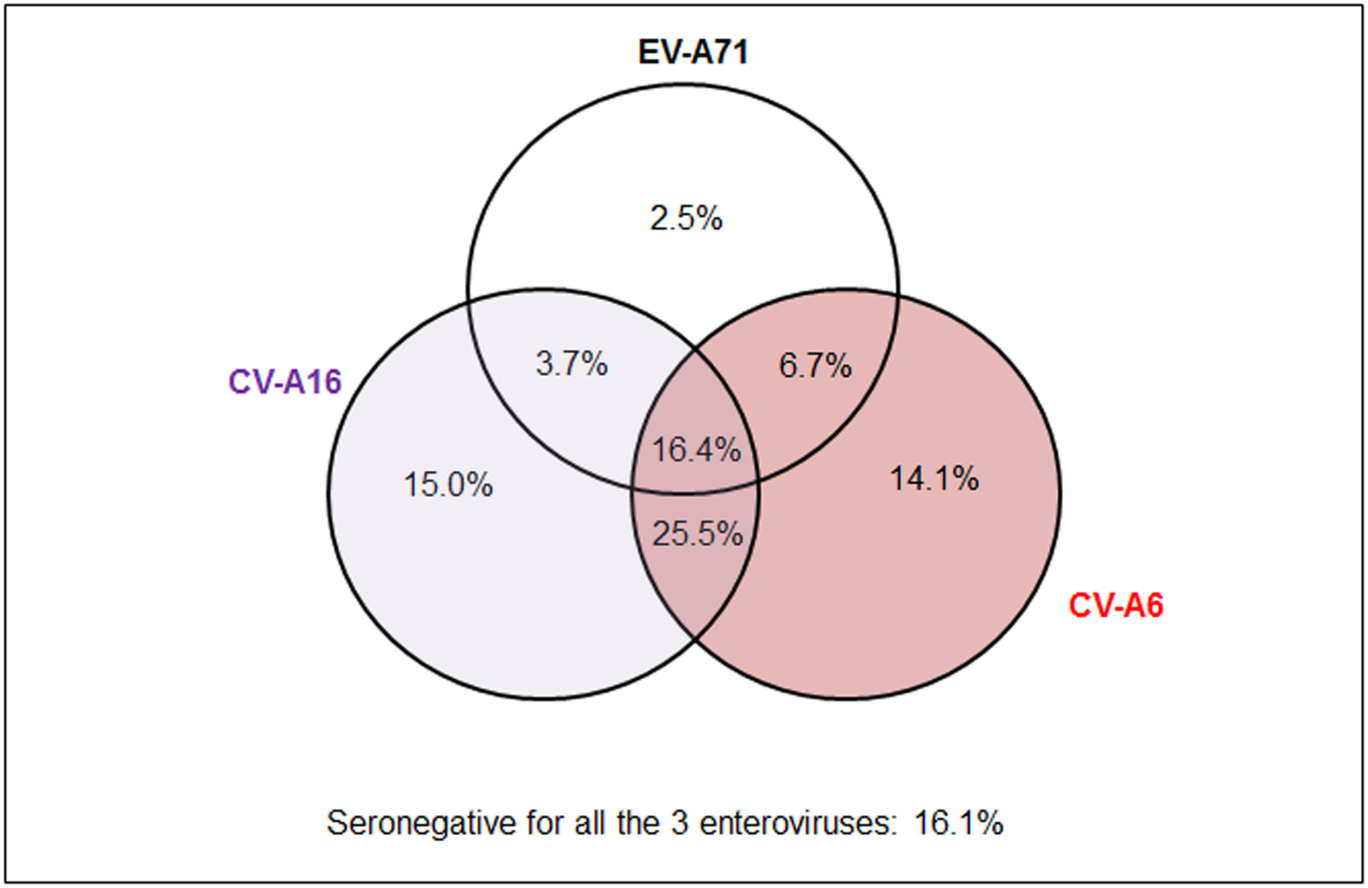
Proportion (%) seropositive for CV-A6, CV-A16 and EV-A71 among 700 subjects aged 1–17 years, Singapore, 2008–2010.

The proportions of subjects who were seropositive for the different enteroviruses are shown in a Venn diagram (Fig 2). Close to one-third (31.6%) were positive for only one enterovirus, while 35.9% and 16.4% were positive for two and all three enteroviruses, respectively. One in six subjects (16.1%) had no NtAb against any of the three enteroviruses.

**Fig 3:**
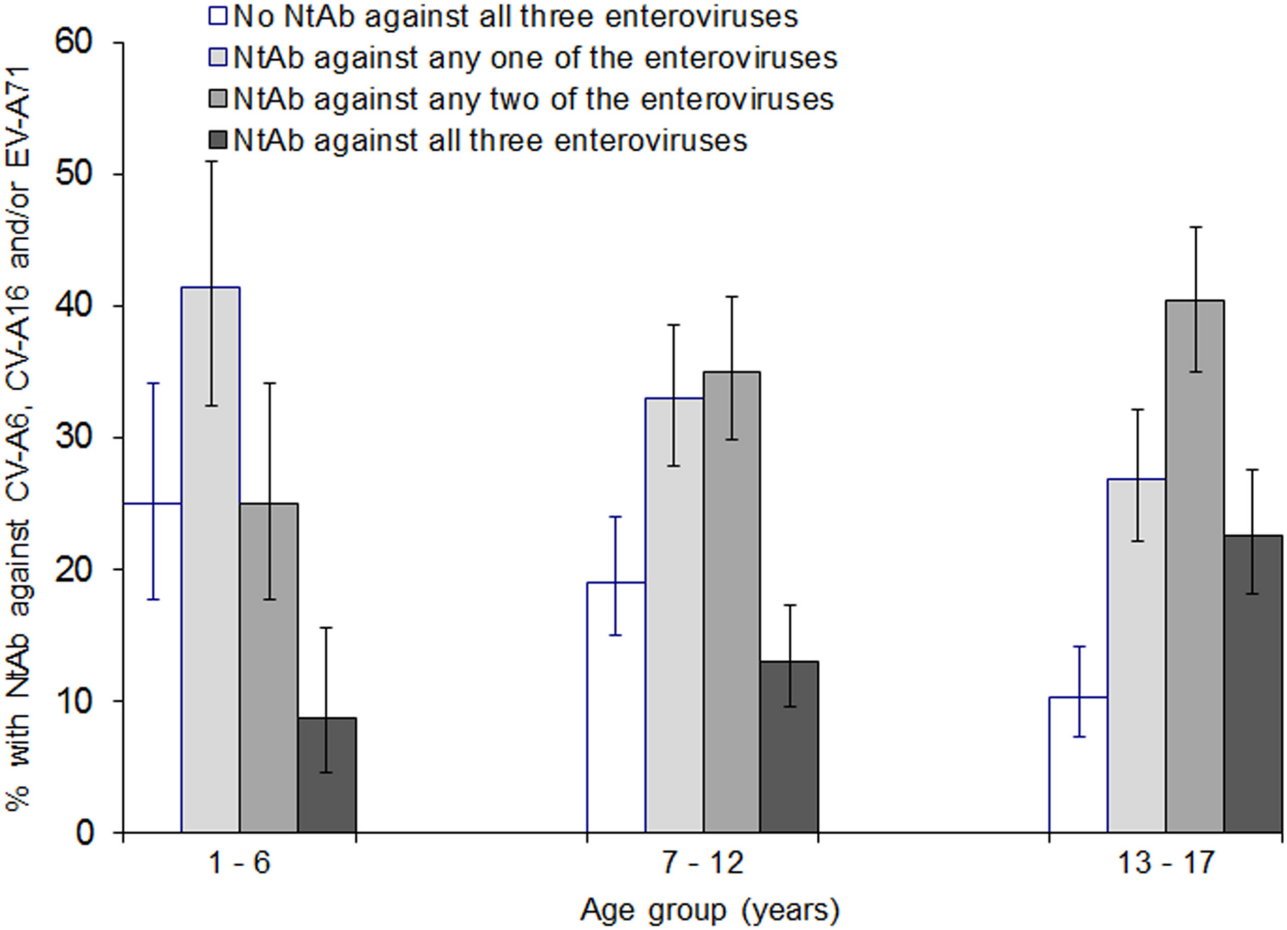
Proportion (%) of subjects with neutralizing antibodies against any one, two or all three enteroviruses, CV-A6, CV-A16 and EV-A71, and those with no immunity (seronegative for all three enteroviruses) by age group, Singapore, 2008–2010. The vertical lines indicate 95% confidence intervals. A subject was defined as immune if NtAb titre was ≥1:8.

The youngest age group of 1–6 years had the highest proportion (25.0%) with no NtAb against any of the three enteroviruses, while the oldest age group of 13–17 years had the highest proportion (22.5%) with NtAb against all three enteroviruses (Fig 3). About two-thirds in each age group had neutralizing antibodies against one or two enteroviruses.

The proportion with NtAb against all three enteroviruses increased significantly with age (*P* < 0.0005). The proportion with NtAb against any two of the enteroviruses also increased significantly with age, whereas the proportion with NtAb against any one of the three enteroviruses decreased significantly with age (both *P* = 0.005).

The proportion with NtAb against at least one of these enteroviruses increased significantly with age, from 75.0% in the 1–6 year age groups to 81.0% in the 7–12 year age group and 89.7% in the 13–17 year age group (*P* < 0.0005).

### Distribution of neutralizing antibody titers among seropositive subjects

For analysis on the level of immunity by NtAb titers, three categories were defined; low (1:8–1:16), moderate (1:32–1:64) and high (1:128–1:1024). There was a difference in the distribution of NtAb levels between EV-A71 and the two coxsackieviruses, CV-A6 and CV-A16. Among subjects aged 1–17 years who tested positive for EV-A71 antibodies, about half (50.2%, 95% CI: 43.5–57.0%) displayed moderate levels of NtAb. Among those who tested positive for CV-A6 and CV-A16 antibodies, over three-quarters had moderate to high NtAb levels (77.8% and 83.6%, respectively). The proportion with high NtAb titers against EV-A71 was lowest at 18.1%, while those against CV-A6 and CV-A16 was more than double at 38.3% and 42.7%, respectively.

**Fig 4:**
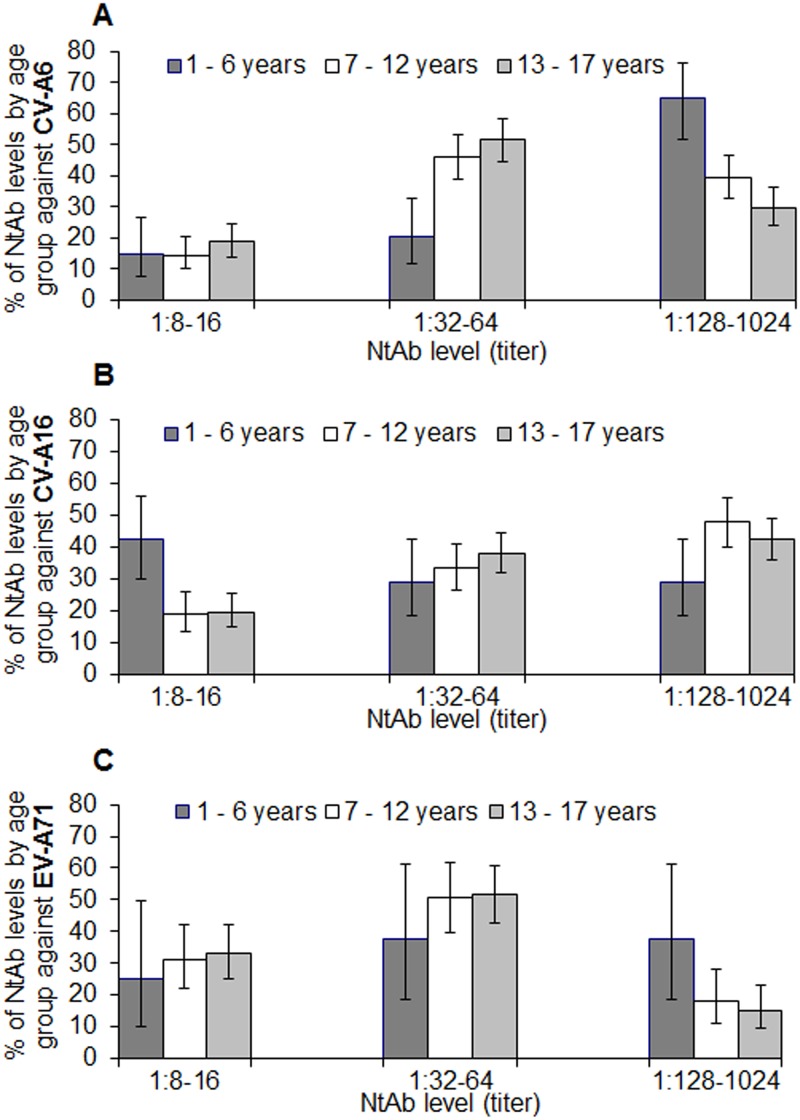
Proportion (%) of seropositive subjects with low, moderate and high levels of neutralizing antibody titres against (A) CV-A6, (B) CV-A16 and (C) EV-A71 by age group, Singapore, 2008–2010. The vertical lines indicate 95% confidence intervals.

For CV-A6, the proportion with high NtAb levels decreased significantly with age (*P* < 0.0005); 64.8% of younger children aged 1–6 years had high NtAb levels compared with 29.8% of adolescents aged 13–17 years (Fig 4A). On the other hand, the proportion of subjects with moderate NtAb levels against CV-A6 increased significantly with age (*P* < 0.0005); the proportion of adolescents aged 13–17 years (51.5%) who had moderate NtAb levels was 2.5 times that of younger children aged 1–6 years (20.4%).

Nearly half (47.7%) the older children aged 7–12 years had high NtAb levels against CV-A16, compared with 28.8% of younger children aged 1–6 years (*P* = 0.018) (Fig 4B). The proportion of subjects with high NtAb levels against EV-A71 was significantly higher in the 1–6 year olds at 37.5%, compared with 15.2% in the 13–17 year olds (*P* = 0.03) (Fig 4C).

### Geometric mean titers in seropositive subjects

Among children and adolescents aged 1–17 years, the GMT of NtAb against CV-A6 and CV-A16 among seropositive samples were 68.4 (95% CI: 61.6–75.9) and 63.2 (95% CI: 56.6–70.6), respectively. It was the lowest at 34.9 (95% CI: 30.7–39.8) for EV-A71.

**Fig 5:**
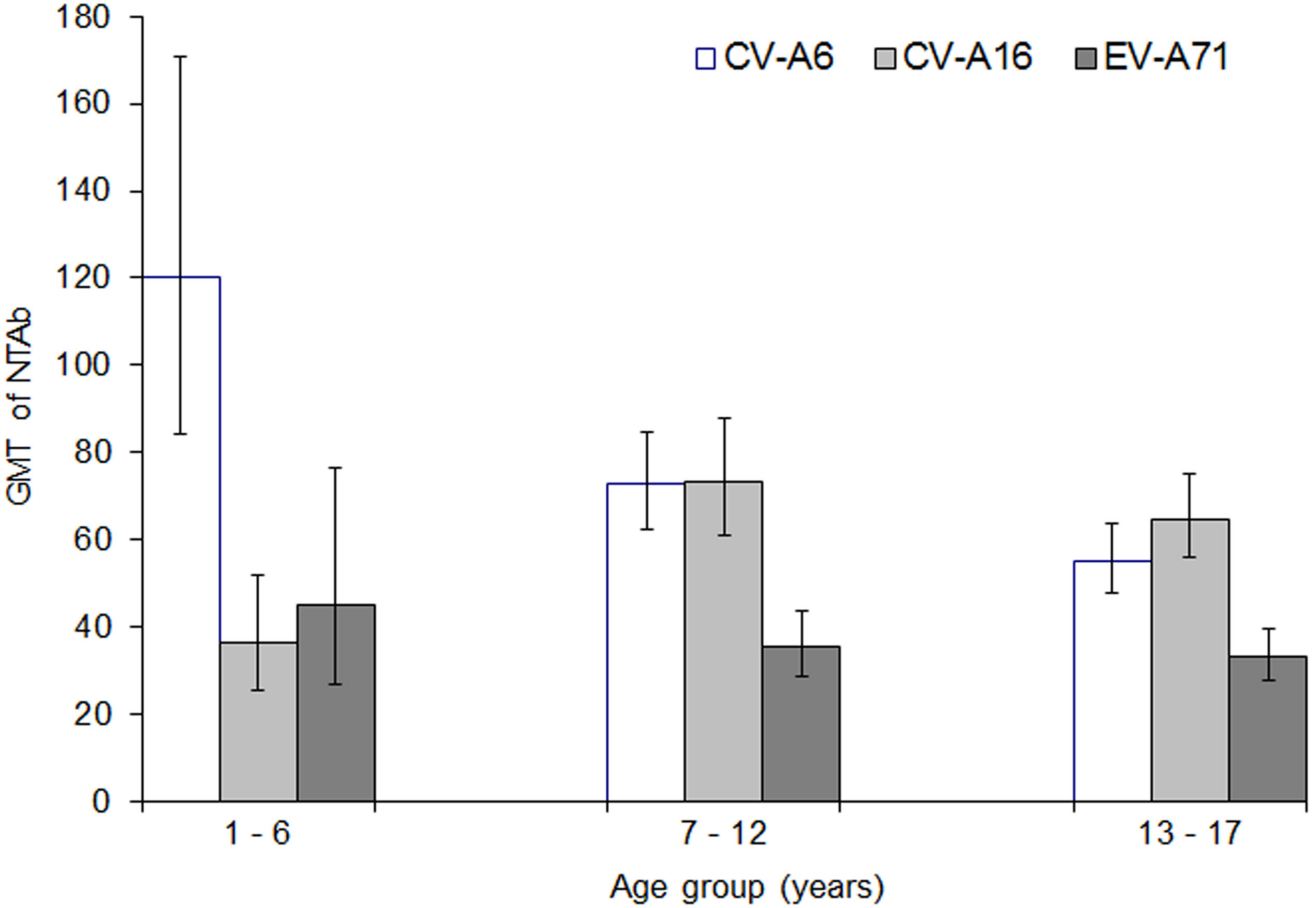
Geometric mean titre (GMT) of neutralizing antibody among seropositive subjects against CV-A6, CV-A16 and EV-A71 by age group, Singapore, 2008–2010. The vertical lines indicate 95% confidence intervals.

The GMT of NtAb against CV-A6 was higher than those against CV-A16 and EV-A71 among pre-school children aged 1–6 years (Fig 5). On the other hand, the GMT of NtAb against EV-A71 was lower than the GMTs against the two coxsackieviruses in the older age groups of 7–12 years and 13–17 years.

A significant declining trend in the GMT of NtAb against CV-A6 was observed across the three age groups (Fig 5); the GMT decreased from 120.0 (95% CI: 84.4–170.8) among pre-school children aged 1–6 years to 72.9 (95% CI: 62.5–84.9) in older children aged 7–12 years and 55.2 (95% CI: 47.9–63.8) in adolescents aged 13–17 years.

For CV-A16, the GMT was significantly lower among younger children aged 1–6 years (GMT 36.6; 95% CI: 25.7–52.0) than in older children aged 7–12 years (GMT 73.3; 95% CI: 61.2–87.9) and adolescents aged 13–17 years (GMT 64.8; 95% CI: 55.9–75.1) (Fig 5). There was no significant difference in the GMT of NtAb against CV-A16 between the two older age groups. No significant differences in the GMT of EV-A71 NtAb by age group were detected.

## Discussion

Our study indicated that CV-A6 and CV-A16 infections are very prevalent among Singapore children and adolescents aged 1–17 years, while EV-A71 infections are less common. The seroprevalence of EV-A71 (29.3%) was half that of CV-A6 (62.7%) and CV-A16 (60.6%). The lower seroprevalence of EV-A71 compared to CV-A16 was also observed in a study in Germany in 2006 [39]. Differences in the seroprevalence of these three enteroviruses reflected their cumulative epidemiology over the past one to two decades, with CV-A6 and CV-A16 more commonly associated with HFMD than EV-A71.

About half (52.3%) of Singapore children and adolescents were seropositive for two or all three enteroviruses, while 83.9% were seropositive for at least one of the enteroviruses. A high proportion of the children (89.7%) had been infected by at least one of the three enteroviruses by the age of 13–17 years (Fig 3). Infection is continually acquired from early childhood to adolescent age; three-quarters of children aged 1–6 years had been infected by at least one of these enteroviruses, which increased to 80.0% in older children aged 7–12 years and 89.7% in adolescents aged 13–17 years. The age-specific prevalence corroborated with the surveillance data on incidence rate and institutional outbreaks of HFMD reported to the MOH. Most of the outbreaks occurred in childcare centers where 1–6 year olds congregated [30]. The numbers of outbreaks reported in kindergartens attended by children aged 5–6 years and in primary schools attended by children aged 7–12 years were much lower. This indicated that young children aged 1–6 years were most susceptible to the three enteroviruses.

The predominant circulating enteroviruses associated with nationwide epidemics alternated between CV-A16 and EV-A71 during the period 2005–2009 (Fig 1). Hence, it was not surprising that 69.8% of children and adolescents had NtAb against at least one of these two enteroviruses (Fig 2). The first serologic survey on EV-A71 in Singapore was conducted among 856 children aged 12 years or younger at a pediatric clinic at NUH from July 1996 to December 1997, before the first EV-A71-associated HFMD epidemic occurred in 2000 [40]. Only 0.8% of children aged 1–23 months were found to have NtAb against EV-A71. The infection was largely acquired at 2–5 years of age with the seropositivity increasing at an average rate of 12% per year in this age group. The seroprevalence reached a steady state at about 50% in older children aged 5–12 years. Despite the HFMD epidemics predominantly associated with EV-A71 in 2006 and 2008, our study showed that the seroprevalence had not increased correspondingly, with only 29.3% of subjects aged 1–17 years seropositive for EV-A71 (Table 2). Moreover, only 18.1% of children and adolescents aged 1–17 years had high NtAb titers ≥ 1:128 against EV-A71, which suggested infrequent exposures to EV-A71 (Fig 4C). The seroprevalence increased by about 11% from children aged 1–6 years to older children aged 7–12 years and by another 11% in adolescents aged 13–17 years.

In a serologic study of 120 children aged 5–15 years in Vietnam between September 2005 and January 2009, the EV-A71 seroprevalence was found to be much higher at 84% [41]. This was nearly three times the EV-A71 seroprevalence of 29.1% found in our study in this age group. In a study of 614 sera samples collected from children below 5 years of age in Shanghai city of China between November 2010 and early April 2011 prior to the HFMD epidemic season, the EV-A71 seroprevalence was 19.9% [42]. In our study, the EV-A71 seroprevalence in the age group of 1–4 years was lower at 11.1%. Stored serum samples collected from healthy children aged 1–9 years in Guangdong province of China from 2007 to 2009 were analyzed to compare the annual levels of NtAb against EV-A71 and CV-A16 in the three-year period from 2007 to 2009 [43]. While the EV-A71 seroprevalence was also higher than that found in our study (9.5% in the same age group), the CV-A16 seroprevalence was comparable with that of our study (61.9%).

Before late 2009, CV-A6 had not been associated with HFMD epidemics in Singapore. HFMD epidemics had occurred annually with two peaks in each year from 2005 to 2009, followed by a single peak since 2010. The emergence of CV-A6 as the predominant circulating enterovirus was associated with a change in the annual pattern of HFMD epidemics from bimodal to unimodal. In our study, about 14.1% were seropositive for only CV-A6 (Fig 2). When CV-A6 first emerged as the predominant enterovirus in 2010, the incidence of HFMD was 608.2 per 100,000 population, nearly twice that in 2009 when CV-A16 was associated with the epidemic in the first half of the year. The accumulation of children susceptible to CV-A6 infection could have triggered the largest HFMD epidemic in the last decade in 2012 (698.8 per 100,000 population) [29]. In our study, the higher GMT of NtAb against CV-A6 compared to those against CV-A16 and EV-A71 in the youngest age group of 1–6 years suggested its recent emergence (Fig 5).

Besides recent HFMD epidemics associated with CV-A6 in Singapore, outbreaks caused by CV-A6 were also reported in other Asian countries, such as Taiwan in 2007 [44], 2009 [44] and 2010 [45], Japan in 2011 [46], Thailand in 2012 [47], and China in 2013 [48]. One postulation for the higher activity of CV-A6 in recent years is the build-up of population immunologically naive to CV-A6 over time, since CV-A16 and EV-A71 infections have been more frequently reported in earlier years. In Singapore, the seroprevalence of CV-A6 was likely to be low in the early 2000s, and this may have led to the recently observed cyclical pattern of HFMD cases since 2010. Hence, it is important to conduct seroprevalence surveys periodically, so as to assess the impact of past epidemics associated with these enteroviruses and to discern any changes in their seroprevalence levels for public health surveillance and control. For example, in Taiwan, a considerable reduction in EV-A71 seroprevalence in 1994 and 1997 compared to that of an earlier period from 1989 to 1993 was deemed to be a harbinger of the large epidemic in 1998 [49].

Malays had the highest seroprevalence of CV-A16 and EV-A71, which were significantly higher compared to that of Chinese against these two enteroviruses. This corresponded to the ethnic-specific incidence rate of HFMD among Singapore residents. Among the three major ethnic groups, Malays had the highest HFMD incidence per 100,000 population, followed by Chinese and Indians, since 2006 [30].

More than 90% of infections caused by non-polio enteroviruses are asymptomatic or result in mild non-specific febrile illness [50]. There have been recent reports of atypical presentation of HFMD associated with CV-A6, including patients who presented with onychomadesis with the loss of nail occurring one to two months after initial symptoms [51]. Severe illnesses such as myocarditis, pericarditis, encephalitis or paralysis are rare [52]. In Singapore, the case fatality rate of HFMD was very low at less than 1 per 15,000 between 2000 and 2010 [32]. A total of eight pediatric deaths were reported during this period; four in 2000 (including two children tested positive for EV-A71), three in 2001 (two tested positive for EV-A71) and one in 2008 (positive for EV-A71). Almost one in 50 notified HFMD cases were hospitalized during the EV-A71-associated HFMD epidemics between March and April 2006 (1.8%), and between April and May 2008 (1.6%). This was more than double the proportion hospitalized during the HFMD epidemics associated with CV-A16 (0.8% between March and April 2005, and 0.7% between April and May 2007) [30]. In comparison, the proportion hospitalized during the HFMD epidemics when CV-A6 was the main circulating enterovirus was lowest at 0.4% between October and November 2009, and 0.5% between July and August 2010. This suggested that while complications of HFMD cases were rare, EV-A71 infections seemed to be more severe compared to CV-A16 and CV-A6. Similar observations had been made in China [18], Taiwan [53,54], Japan [15] and Vietnam [24].

The study design of the NPSS 2008–2010 was determined after careful considerations of the advantages and disadvantages of population-based sampling versus laboratory-based sampling [32]. To minimize potential selection bias in the survey, sera of patients who had been diagnosed with HFMD were excluded from our study. National serologic studies using residual sera from diagnostic laboratories had been carried out to estimate EV-A71 seroprevalence in Singapore [32], Germany [39] and Vietnam [41]. Although the serum samples were collected several years ago, our retrospective study can provide valuable information to gauge the extent of herd immunity in the pediatric community against these clinically important enteroviruses. Ideally, if conducted at regular intervals by countries in South-east and East Asia with sharing of the findings, such seroepidemiologic surveys can aid in the prediction and/or mitigation of future HFMD outbreaks in the region. Given that recent clinical trials of EV-A71 vaccines have yielded encouraging data on efficacy and safety, these serologic surveys may have also added significance in the long run when there are commercial vaccines available in future [55].

The interpretation of polyclonal antibodies in sera is complex and challenging as we do not have information on the subjects’ past history of subclinical or clinical enteroviral infections in this cross-sectional seroprevalence study. Another possible limitation of our study is the potential cross-reactivity of EV-A71 NtAb against CV-A16 [56,57]. On the other hand, there had been studies showing no or weak cross-reactivity of EV-A71 NtAb against CV-A16, which may be partially explained by the differences in their VP1 amino acid sequences [58–60].

In our study, a subgenotype B4 strain was used in the neutralization test for EV-A71, which was predominant during the 2000 epidemic in Singapore. The predominant EV-A71 subgenotype was B5 during the 2006 epidemic [30] and 2008 epidemic [31]. While it is not known whether different EV-A71 genotypes are uniformly neutralized by “standard” polyclonal immune sera, it is likely to be the case based on epidemiologic evidence in Vietnam that there had not been any HFMD cases found to have recurrent infections associated with EV-A71 [41]. There may also be concern about the potential for cross-reactivity of neutralizing antibodies among different EV-A71 genotypes and subgenotypes [61–63]. However, we and others have demonstrated that the EV-A71 NtAb assay using the subgenotype B4 strain can detect EV-A71 specific cross-neutralizing antibodies regardless of subgenotype [64,65].

Our findings shed light on the immunologic response of the general pediatric population after the occurrence, since 2000, of HFMD epidemics associated with CV-A6, CV-A16 and EV-A71, and provided a direct comparison of the seroprevalence of these three enteroviruses in the same group of study subjects. In this study, we found that 83.9% had NtAb against at least one of the three enteroviruses, which indicated that these infections are very common in children and adolescents in Singapore.
